# Pennsylvania Jersey-Breeders’ Association

**Published:** 1898-04

**Authors:** 


### PENNSYLVANIA JERSEY-BREEDERS' ASSOCIATION.

The Pennsylvania Jersey Breeders' Association met in Pittsburg, March 17th. Among the sixty dairymen present were four veterinarians: Dr. George B. Jobson, of Franklin, President of the State Veterinary Medical Association; Dr. J. C. McNeil, of Pittsburg; Dr. Leonard Pearson, State Veterinarian, and Dr. N. Rechtenwald, Pittsburg. Dr. Jobson read a very interesting paper on "Why Cows Do Not Breed." This paper was discussed by Dr. Rechtenwald, who exhibited some very useful, original instruments for dilating the cervix. Dr. Pearson lectured on the subject of tuberculosis of cattle. And that his views and work were not distasteful to the Jersey breeders is shown by the fact that he was afterward elected an honorary member of the Association.

